# Non-functional, non-mutated multifocal neuroendocrine neoplasms in a postpartum female: a Case Report of an infrequent disease

**DOI:** 10.3389/fmed.2025.1619565

**Published:** 2025-11-17

**Authors:** Fangmei Ling, Wenkai Yang, Jinhua Wu, Jing Li

**Affiliations:** Department of Gastroenterology, The People’s Hospital of Guangxi Zhuang Autonomous Region, Nanning, China

**Keywords:** neuroendocrine neoplasms, pregnancy, early detection, non-functional, metastability, abdominal pain

## Abstract

**Background:**

Neuroendocrine neoplasms (NENs) represent a relatively rare yet heterogeneous group of neoplasms arising from diverse anatomical origins. The inherent variability in clinical manifestations and gradations of biological aggressiveness pose substantial challenges in diagnostic and therapeutic management. This report presents a diagnostically complex case of postpartum non-functional NENs.

**Case presentation:**

A 39-years-old female with childhood history of acute pancreatitis presented with intermittent abdominal pain and considered pancreatitis during pregnancy. Initial laboratory evaluation revealed elevated carcinoembryonic antigen levels with normal gastrin, serum glucose, and electrolyte profiles. Contrast-enhanced CT and MRI demonstrated multifocal lesions involving the left pulmonary lobe, pancreatic tail, thyroid gland, appendix, bilateral adnexa, and vertebral bodies. Diagnostic endoscopic evaluation identified a raised lesion at the appendiceal orifice, while bronchoscopic visualization revealed neoplastic obstruction in the lingular segment of the left upper lobe. 68Ga-DOTANOC PET/CT confirmed widespread somatostatin receptor-negative lesions (excluding thyroid nodules), suggesting receptor heterogeneity. Comprehensive genomic profiling failed to identify clinically actionable mutations. Histopathological analysis of biopsy specimens established two distinct primaries: pulmonary atypical carcinoid (Ki-67 proliferation index ∼30%) and appendiceal neuroendocrine neoplasm (WHO G2, Ki-67 ∼5%). Following multidisciplinary tumor board consensus, the patient was diagnosed with synchronous primary NETs (pulmonary and appendiceal origin) with multisystem metastases, initiating CAPTEM chemotherapy regimen.

**Conclusion:**

Synchronous non-functional NENs with metastases remain exceptionally rare in clinical practice. The predominant presentation with non-specific abdominal pain significantly amplifies diagnostic complexity in such cases. This underscores the necessity for heightened clinical vigilance for NENs when evaluating atypical presentations.

## Introduction

Neuroendocrine neoplasms (NENs) are rare malignancies arising from diffusely distributed neuroendocrine cells, exhibiting heterogeneous phenotypes, morphological diversity, and variable clinical courses. Despite being documented over a century ago, NENs remain underrecognized in clinical practice. The 2022 WHO Classification establishes a refined diagnostic paradigm, categorizing NENs by histomorphology and grading parameters: well-differentiated neuroendocrine tumors (NETs) with low proliferative activity versus poorly differentiated neuroendocrine carcinomas (NECs), which demonstrate aggressive biology. Poorly differentiated NENs typically exhibit reduced neuroendocrine marker expression, accompanied by elevated proliferative indices (Ki-67) and mitotic counts (/2 mm^2^) (1). Differentiating G3 NETs from NECs proves challenging using proliferative indices alone, necessitating integrated assessment of cytomorphology, architecture, and immunohistochemical profiles (2). Well-differentiated NETs characteristically display “salt-and-pepper” chromatin and organoid growth, while NECs often show scant or occasionally abundant cytoplasm. Essential IHC markers include somatostatin receptors (SSTR)2/5, TP53, Rb, Menin, p27, ATRX, and DAXX (3).

Neuroendocrine neoplasms demonstrate low population prevalence (global incidence ∼0.7/100,000), with Chinese data showing 1.14/100,000 (4, 5). Surveillance studies note increasing detection rates (4–7), though metastatic frequency remains stable (0.63–0.69/100,000 person-years) (8). Poor prognosis correlates with advanced age, poor differentiation, and distant metastasis (4). Early detection remains challenging due to non-tissue-specific origin and multi-organ involvement (gastrointestinal, bronchopulmonary, thyroid, vertebral). While most cases are sporadic, 7%–10% demonstrate hereditary predisposition via MEN syndromes (9). This report presents a diagnostically complex case of multifocal NENs with lymphatic dissemination in a postpartum patient, notable for non-syndromic presentation despite extensive tumor burden. The documentation aims to expand evidence for atypical NEN phenotypes and refine diagnostic algorithms for complex presentations.

## Case description

A 39-years-old female presented to the Gastroenterology Department with recurrent postprandial epigastric pain persisting for 11 weeks, including two acute exacerbations after heavy meals. Clinical manifestations included diminished bowel frequency (2–3 spontaneous evacuations/week), acid reflux, and persistent eructation, without concomitant fever, diarrhea, jaundice, cough, or neurological deficits. Her symptoms initially developed during the third trimester of pregnancy (5 weeks pre-delivery) and acutely worsened 9 days after an uncomplicated vaginal delivery. Initial contrast-enhanced abdominal computed tomography (CT) at an outside facility demonstrated partial pancreatic tail necrosis, prompting a provisional diagnosis of acute pancreatitis. While conservative management provided partial relief, persistent abdominal discomfort prompted referral to our institution at 6 weeks postpartum (see Supplementary Figure 1 for diagnostic timeline). Medical history included resolved childhood acute pancreatitis and a significant family history of maternal lymphoma and grandmother’s pancreatic carcinoma.

Physical Examination revealed a firm, minimally mobile 3 mm × 4 mm lymph node along the right sternocleidomastoid border and a non-tender 2 cm × 3 cm right thyroid nodule with smooth contours. Abdominal and thoracic examinations were unremarkable.

Initial laboratory evaluation demonstrated unremarkable routine hematologic, metabolic, and immunologic parameters. Notable elevations included lipase (225 U/L; reference: 0–67 U/L), carcinoembryonic antigen (11.30 μg/L; reference: 0–5.0), cytokeratin 19 fragment (6.33 μg/L; reference: 0–3.30), neuron-specific enolase (56.30 μg/L; reference: 0–16.3), and procalcitonin (2.42 ng/mL; reference: 0–0.05).

**FIGURE 1:**
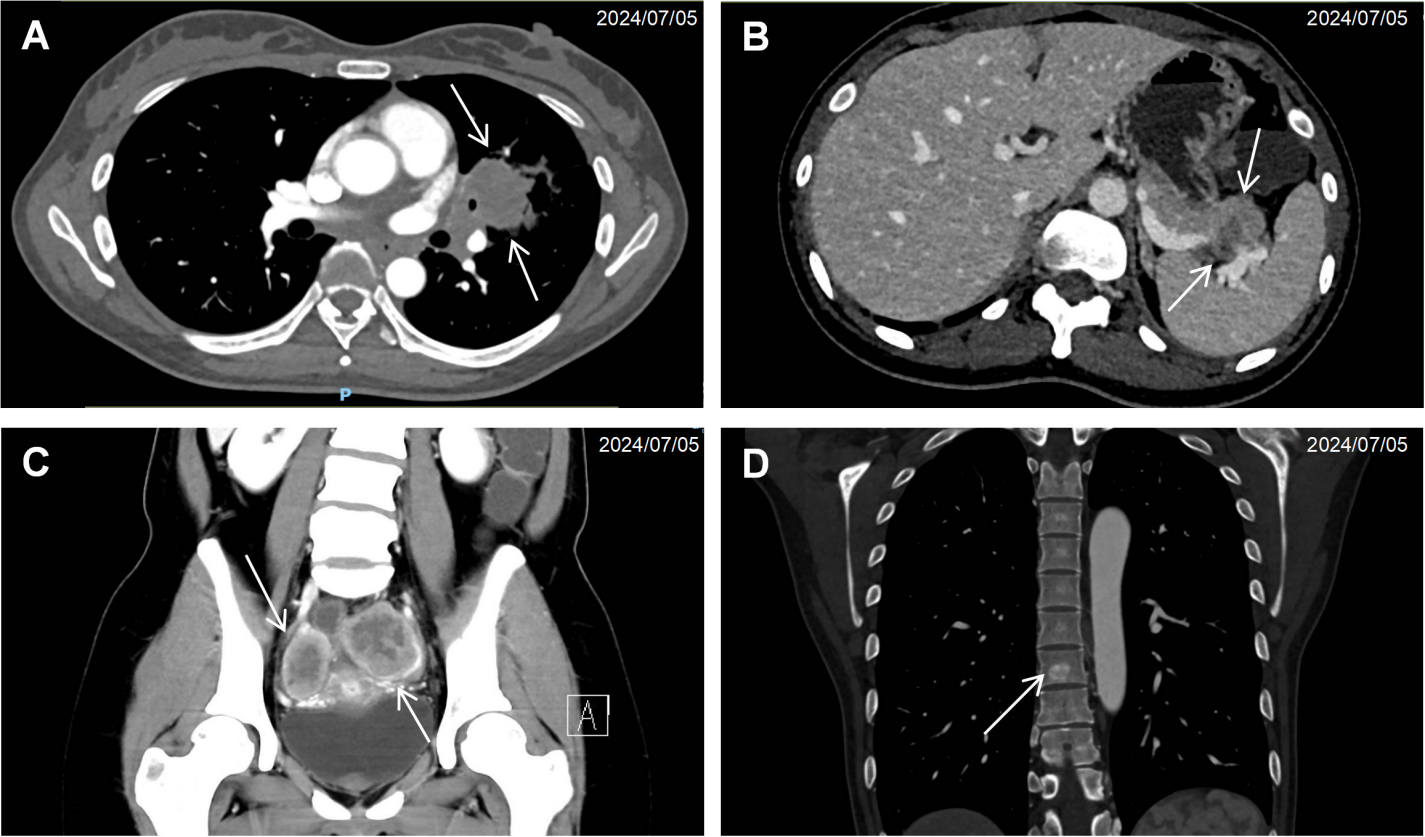
Cross-sectional imaging findings of neuroendocrine neoplasms. **(A)** Contrast-enhanced CT demonstrating a solid pulmonary mass (arrow) in the left upper lobe with associated bronchial truncation. **(B)** Hypodense pancreatic tail lesion (arrow) exhibiting ill-defined margins and poor demarcation from adjacent pancreatic parenchyma. **(C)** Bilateral adnexal complex cystic-solid lesions (arrow). **(D)** Anodular hyperdense lesion (arrow) within the T8 vertebral body.

**FIGURE 2:**
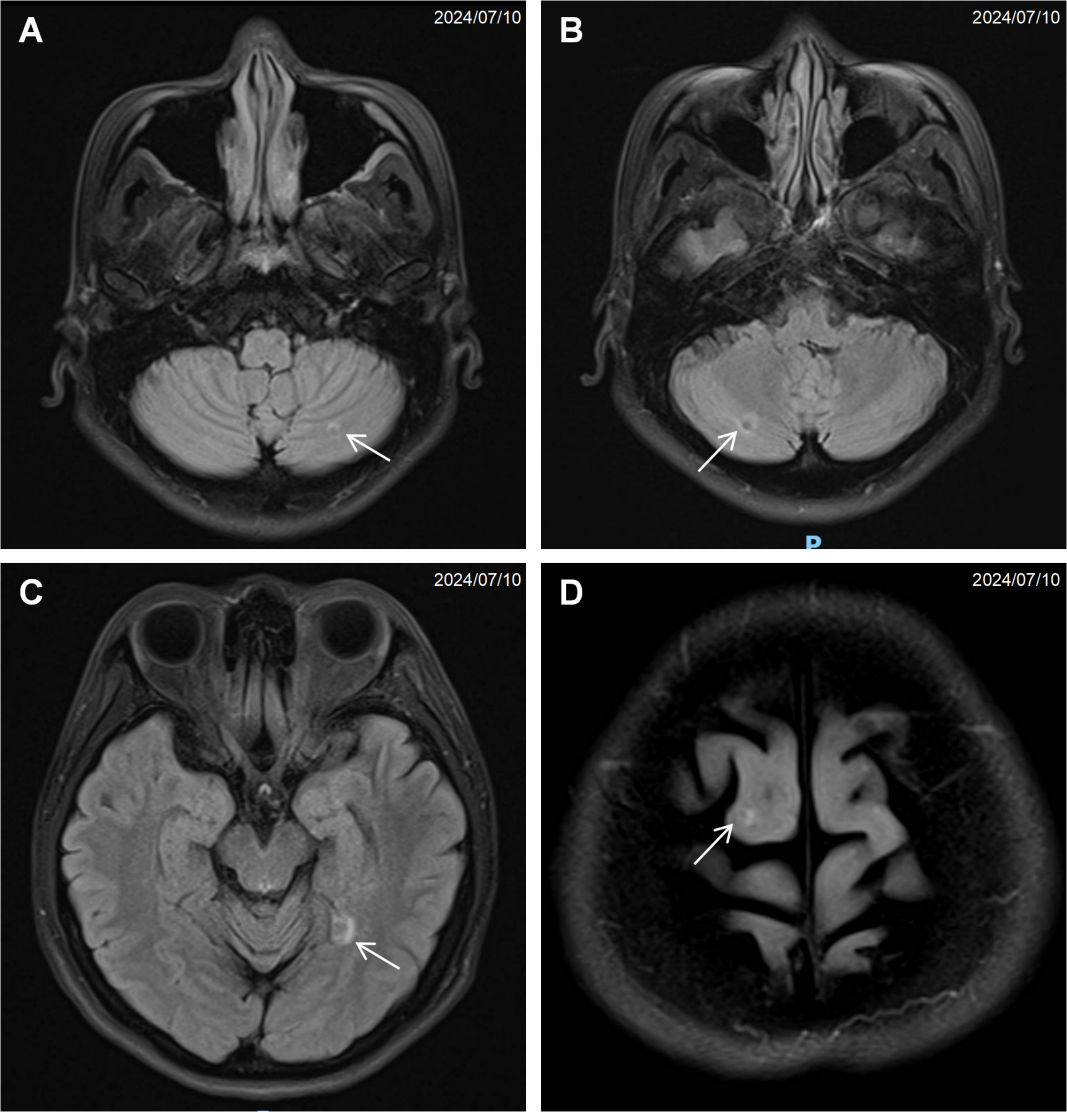
Magnetic resonance imaging (MRI) of the brain showing multiple small nodular signal abnormalities: **(A)** left cerebellar hemisphere (arrow), **(B)** right cerebellar hemisphere (arrow), **(C)** left temporal lobe (arrow), **(D)** right frontal lobe (arrow).

Computed tomography of the chest, abdomen, and pelvis revealed multifocal lesions involving the left lung, pancreas, appendix, and bilateral adnexa (Figure 1). A solid left upper lobe mass (40 × 34 × 46 mm) demonstrated heterogeneous enhancement with bronchial cutoff and pleural indentation. Pancreatic lesions included hypodense masses in the uncinate process (20 mm × 18 mm; progressive enhancement) and tail (28 mm × 22 mm; heterogeneous enhancement). An appendiceal nodule (13 mm × 9 mm) showed uniform enhancement with ileocecal lymphadenopathy. Complex cystic-solid adnexal lesions were observed, largest in the left ovary (55 × 52 × 52 mm). Differential diagnosis included primary lung adenocarcinoma and NET, with additional lesions noted in the thyroid, left breast, and T8/L5 vertebrae. Magnetic resonance imaging (MRI) of the chest and abdomen confirmed multi-organ involvement (left lung, pancreas, bilateral adnexa) with iso- to hyperintense signals. Vertebral MRI demonstrated a T8 nodule (14 mm × 8 mm) with long T1/T2 signals. Cranial MRI showed normal pituitary architecture but abnormal foci in cerebellar hemispheres, left temporal lobe, and right frontal lobe, the largest (4 mm) in the left frontal lobe (Figure 2). Thyroid ultrasound revealed C-TIRADS 4A nodules, with the largest measuring 29 × 22 × 15 mm and showing heterogeneous enhancement on contrast-enhanced ultrasound.

These concerning imaging findings prompted further serological evaluation, which revealed markedly elevated levels of human epididymis protein 4 (HE4; 4169.0 pmol/L, reference: 0–68.9) and calcitonin (48.52 pg/mL, reference: 0–9.8). Several lung cancer-associated antibodies were also elevated: p53 antibody (43.1 U/mL, reference: 0–13.1), PGP9.5 (34.2 U/mL, reference: 0–11.1), SOX2 (18.4 U/mL, reference: 0–10.3), GAGE7 (48.3 U/mL, reference: 0–14.4), and GBU4-5 (13.7 U/mL, reference: 0–7.0). Laboratory values for glucagon, pro-BNP, thyroid function, cortisol, adrenocorticotropic hormone, parathyroid hormone, gastrin-17, and anti-Müllerian hormone were within normal limits. Chromogranin A (CgA) and 24-h urinary 5-hydroxyindoleacetic acid testing were not performed.

**FIGURE 3:**
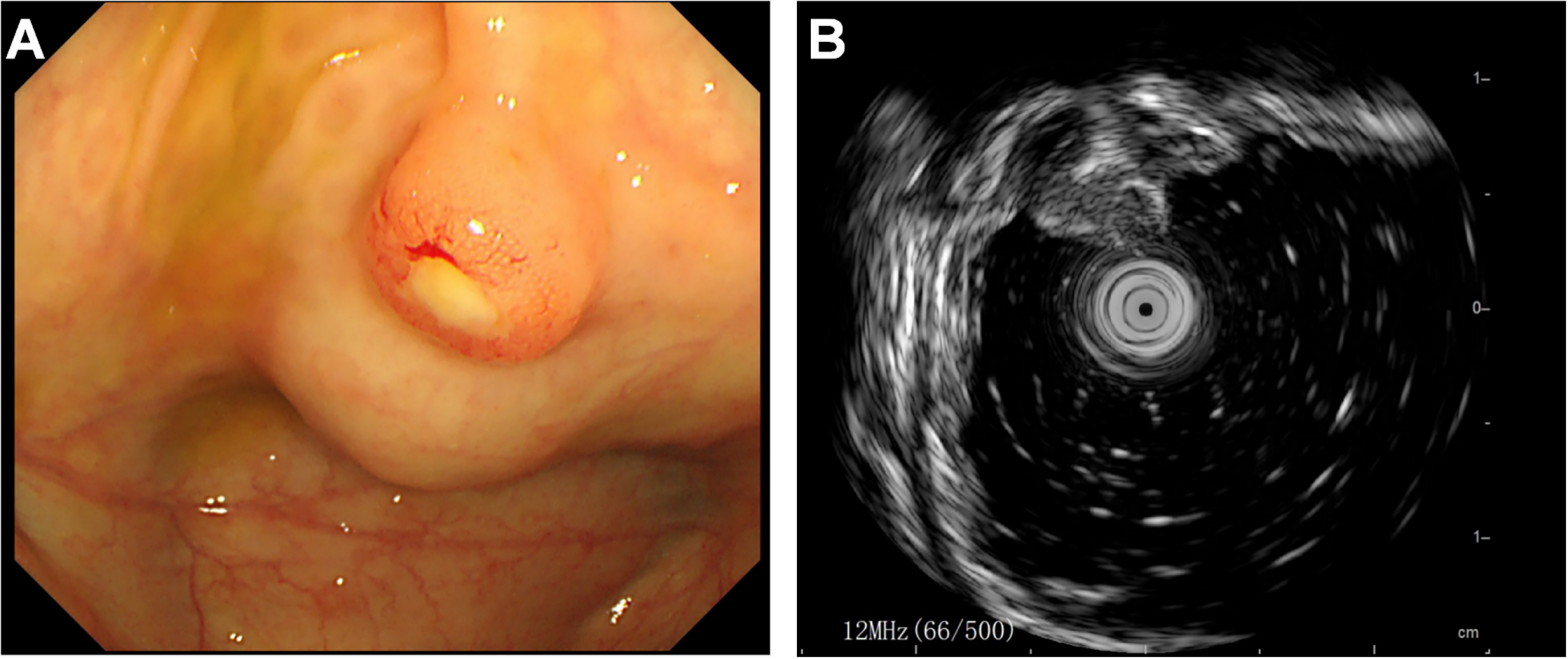
Endoscopic evaluation of appendiceal neuroendocrine tumor. **(A)** Colonoscopic visualization of a hemispherical protrusion at the appendiceal orifice. **(B)** Endoscopic ultrasound revealing a heterogeneous predominantly hypoechoic lesion (calipers) originating from the submucosal layer.

**FIGURE 4:**
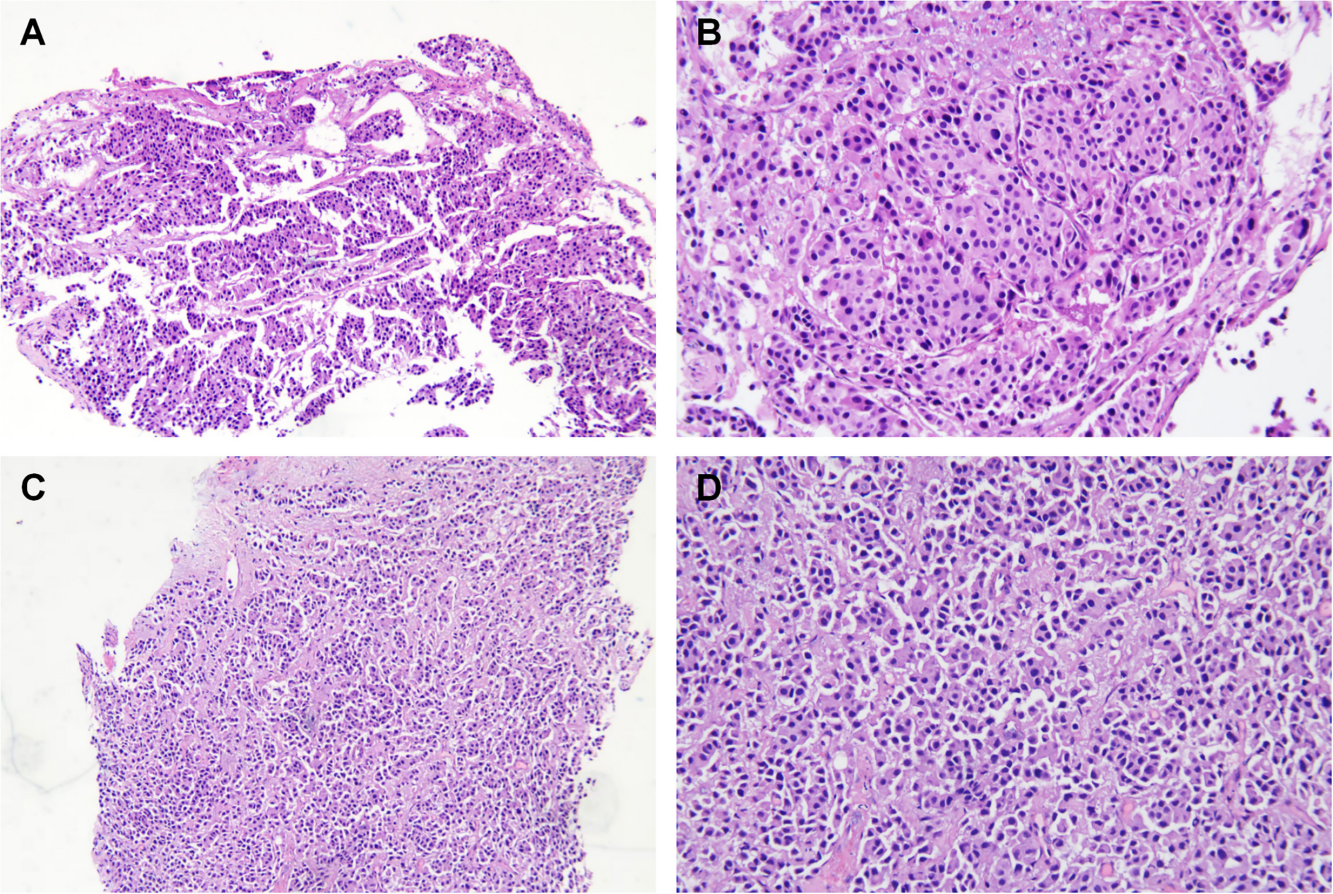
Histopathology of the lung **(A,B)** and appendix tissue **(C,D)**. **(A,B)** H&E staining demonstrating organoid architecture with nest-like growth patterns. Tumor cells exhibit enlarged hyperchromatic nuclei and scattered mitotic figures. **(C,D)** Epithelioid cell infiltration within the lamina propria, arranged in sheet-like and nested clusters (H&E staining). Note abundant cytoplasm and irregular nuclear contours. Left panel: 40× magnification; right panel: 100× magnification.

Subsequently, the patient underwent endoscopic evaluation. Endoscopy demonstrated chronic gastritis, gastric polyps, and duodenal papillae enlargement, with histopathology confirming benign glandular polyps. Endoscopic ultrasound (Figure 3) identified a 6.4 mm × 5.5 mm heteroechoic submucosal lesion protruding from the appendiceal orifice. Biopsy confirmed a neuroendocrine tumor (Figure 4) with immunohistochemical profile (G2 grade, Ki-67 ∼5%): SSTR2(+), Rb(+), P53 (wild expression pattern), Melan-A(−), HMB45(−), CD56(+), CgA(+), S100(−), SOX-10(−), Syn(+), E-cad(cell membrane positive), CEA[P](+), CEA[M](+), CK(+). Bronchoscopy demonstrated neoplastic obstruction in the left lower lingular segment with ultrasonographically evident lymph node clusters (stations 7/11). Histopathology (Figure 4) confirmed atypical carcinoid tumor (Ki-67 ∼30%) exhibiting CK(+), CgA(+), Syn(+), CK7(+), CD56(+), TTF-1(8G7G3/1)(+), with negative SSTR2 and lineage-specific markers. The station 7 and 11 Lymph node biopsies confirmed metastatic atypical carcinoid. Cytologic analysis revealed moderate cellular atypia without infectious organisms.

Further evaluation with ^68^Ga-DOTANOC PET/CT imaging demonstrated heterogeneous distribution of somatostatin receptor (SSTR) affinity. A soft tissue mass near the left hilum showed mild radiotracer uptake (SUVmax 1.9). No significantly increased radiotracer uptake was observed in the slightly thickened pancreatic head, the appendix, the bilateral adnexal soft tissue shadows, the osteosclerotic lesions at T8/L5 vertebrae, or the enlarged left supraclavicular lymph node. No abnormal masses or elevated SSTR expression was detected in the bilateral breast regions or brain. In contrast, both thyroid nodules demonstrated markedly increased avidity (SUVmax 12.0), with the larger right nodule (20 mm) showing high SSTR expression.

Genetic analysis identified no pathogenic variants in MEN1, NF1, RET, or VHL. Missense mutations in NOTCH4 and STK11 were classified as germline variants of uncertain significance.

Following multidisciplinary review, the patient was diagnosed with multifocal NENs (pulmonary atypical carcinoid and appendiceal NET G2) with metastases to pancreas, thyroid, adnexa and vertebrae. The CAPTEM regimen was initiated as follows: capecitabine 1000 mg twice daily on days 1–14 and temozolomide 300 mg on days 10–14, on a 28-days cycle. At 3-months follow-up, the patient reported no significant treatment-related toxicity. Imaging demonstrated a mixed response: reduction in left pulmonary mass (27 × 30 × 26 mm), pancreatic tail lesion (19 mm × 19 mm), and appendiceal nodule (10 mm × 8 mm), but progression of bilateral adnexal lesions (largest 57 × 58 × 50 mm). Thyroid and vertebral lesions remained stable. The patient subsequently transferred care abroad, limiting further follow-up.

## Discussion

Neuroendocrine neoplasms, first documented in the early 20th century, remain diagnostically challenging due to their low incidence and heterogeneous presentations. We report a reproductive-age female with multifocal non-functional NENs presenting with pancreatitis-like symptoms, no germline mutations, and complex diagnostic features, highlighting barriers to early recognition.

Neuroendocrine neoplasms demonstrate pan-organ tropism, predominantly involving the gastroenteropancreatic (GEP) system (55%–70% of cases), lungs, ovaries, uterus, skin, biliary tract, and thymus (4, 10). This case featured primary lung and appendiceal lesions with metastases to pancreas, thyroid, adnexa, and vertebrae. Geographic variation in primary site distribution is well-established: Chinese cohorts show pancreatic predominance (33.5%), contrasting with U.S. data where pulmonary and small intestinal primaries prevail (19.8% and 16.6%, respectively) (5). Western populations exhibit higher small bowel NEN incidence, suggesting gene-environment interactions (8, 11). Our patient’s Asian ancestry with prolonged U.S. residency may reflect such influences.

Most NENs are sporadic with poorly understood etiology, though a subset demonstrates hereditary predisposition as multiple endocrine neoplasia (MEN) syndromes (12). MEN is classified into four molecularly defined subtypes (MEN1-4) based on involved endocrine organs and genetic evidence (9). MEN1 (menin mutations) and MEN2 (RET alterations) represent most cases; rarer associations include VHL, NF1, and TSC (13).

This case’s multiorgan involvement and familial cancer history raised suspicion for MEN syndrome. NENs show site-specific molecular patterns: pulmonary large-cell NECs demonstrate FGF2 alterations; atypical carcinoids harbor KIT/PTEN/HNF1A/SMO variants; and GEP-NENs exhibit grade-dependent heterogeneity – low-grade tumors show ATRX/ARID1A/MEN1 aberrations while high-grade lesions display TP53/KRAS/APC alterations (14–16). Comprehensive germline testing identified no pathogenic variants in MEN1, NF1, VHL, or RET, but revealed VUS in NOTCH4 and STK11 – the latter documented in pulmonary large-cell NEC (17). This indication underscores the imperative for universal genetic counseling in multifocal NENs presentations.

Synchronous NENs of distinct primary origins are rarely reported outside MEN1 syndrome. This case of appendiceal and pulmonary NENs without MEN1 mutations represents a diagnostically distinct entity. While appendiceal NENs are typically indolent (18), the pulmonary component was an atypical carcinoid (AC)–an aggressive subtype (<1% of lung cancers) with frequent nodal involvement and metastatic potential (19, 20). AC demonstrates inferior survival (3-/5-/10-years: 86.0%/74.3%/57.8%) versus typical carcinoids (98%/90.0%/73.7%) (20). Nearly 20% of AC patients present with metastatic disease (19), commonly involving lung, liver, bone, pleura, and pericardium–consistent with the aggressive course observed here.

This case represents synchronous primaries–pulmonary AC and appendiceal NET G2. Discordant SSTR expression and histomorphology support multifocal origin rather than metastatic spread. Such heterogeneity may reflect independent clonal evolution or epigenetic diversification (21, 22), as evidenced by distinct allelic loss patterns in multifocal intestinal NENs (22). Spatial heterogeneity in SSTR2/Ki-67 can occur within single tumors (23), exemplified here by SSTR avidity limited to the thyroid lesion. These findings emphasize the need for multi-site biomarker profiling to guide therapy, though sampling constraints prevented histologic confirmation of all radiologic lesions.

A critical diagnostic consideration in this case revolves around the temporal association between late gestational symptom onset and postpartum NEN diagnosis, raising questions about pregnancy-specific pathophysiological contributors. Gestational hormone surges may influence tumorigenesis, though ethical exclusion of pregnant patients from oncology trials limits epidemiological evidence. Hormonally responsive NENs may express estrogen receptors (ERα/ERβ) and luteinizing hormone/choriogonadotropin receptors (24, 25). A UK cohort reported postpartum progression in 35% of gestational NETs, with ER-positive tumor cells confirmed in three progressive cases (26). Elevated estrogen, progesterone, hCG, IGF-1, and leptin may promote proliferation via PI3K/AKT/mTOR signaling and epigenetic mechanisms (27), supporting biological plausibility in this case.

Given that the patient’s symptoms emerged in the late stages of pregnancy, albeit without a definitive NENs diagnosis at that time, a brief discussion on diagnostic approaches in this specific patient population is warranted. Pregnancy-compatible imaging (serial ultrasonography and non-contrast MRI) is prioritized over radiation-based modalities (28). Serologic and immunohistochemical profiling provide critical adjunct data. Chromogranin A, while non-specific, remains the best-validated circulating biomarker (sensitivity 43%–100%; specificity 10%–96%) (29–31). Complementary markers include synaptophysin, SSTR expression, CD56, and 5-HIAA (32). Our case demonstrated appendiceal (SSTR2+/CgA+/Syn+/CD56+) and pulmonary (SSTR2−/CgA+/Syn+/CD56+) profiles, confirming multifocal neuroendocrine differentiation despite the absence of serum CgA quantification.

It should also be noted that histopathological findings did not fully correlate with the 68Ga-DOTANOC PET/CT imaging results (33). The absent SSTR avidity despite positive SSTR2 immunohistochemistry in the appendiceal lesion may arise from spatial resolution limitations of 68Ga-PET/CT (34), detection of sub-threshold receptor expression by IHC (35), or sampling bias from tumor heterogeneity. These factors underscore the need for correlative multimodal assessment in NEN diagnosis.

Thorough history and physical examination constitute the diagnostic foundation for neuroendocrine tumors. This case demonstrates how diagnostic delays result from both gestational status and non-specific presentations. While pulmonary NETs typically cause respiratory symptoms (36), this patient’s confirmed lung and appendiceal lesions lacked site-specific manifestations, illustrating the diagnostic complexity of non-functional variants. Functional NETs (20%–40% of cases) produce characteristic hormonal syndromes via peptide secretion (10, 37, 38), whereas non-functional NETs (60%–80%) typically remain asymptomatic until mass effects or metastatic complications develop. This patient showed no biochemical or clinical evidence of hormonal hypersecretion. The absence of paraneoplastic features highlights the imperative for multimodal evaluation–integrating histopathology (discordant Ki-67 indices), molecular profiling (SSTR2 heterogeneity), and advanced imaging–in detecting occult multifocal disease. Such complexity necessitates systematic review through multidisciplinary tumor boards.

Neuroendocrine neoplasms are managed with goals of symptom control, disease stabilization, and quality of life preservation. Surgical resection remains curative for localized or locoregional disease, while selected metastatic cases may benefit from primary tumor resection to alleviate hormonal symptoms or reduce tumor burden (39). Treatment selection is guided by symptom profile, somatostatin receptor (SSTR) expression, tumor origin, and histologic grade. First-line somatostatin analogs (SSAs) provide both antisecretory and antiproliferative effects for SSTR-positive tumors or carcinoid syndrome (12, 37), with emerging utility in indolent pulmonary carcinoids (40). Peptide receptor radionuclide therapy (PRRT) offers targeted radiotherapy for SSTR-positive disease, though pulmonary NET efficacy remains under investigation (41). The mTOR inhibitor everolimus demonstrates efficacy in SSA-refractory pulmonary or gastroenteropancreatic NENs with median PFS of 16.4 months versus 11.3 months for placebo, though requires monitoring for pneumonitis (13% incidence) (42, 43).

The CAPTEM regimen (capecitabine plus temozolomide) was selected through multidisciplinary consensus based on the patient’s multifocal, non-functional neuroendocrine neoplasms with heterogeneous SSTR expression. While somatostatin analogs represent first-line therapy for SSTR-positive disease, the coexistence of SSTR-negative metastases–particularly the pulmonary lesion–rendered SSA monotherapy suboptimal. Temozolomide-based chemotherapy is established as a cornerstone strategy for pulmonary NETs. The 2021 European Neuroendocrine Tumor Society guidelines position temozolomide and platinum-based therapies (e.g., oxaliplatin) as salvage options for everolimus-resistant cases (41). The CAPTEM combination provides mechanistic synergy through temozolomide-induced DNA damage (O6-methylguanine lesions) and capecitabine-mediated thymidylate synthase inhibition, particularly relevant for tumors with aggressive biology (Ki-67 ≥ 30%).

This approach is supported by accumulating clinical evidence. Real-world data from Crespo et al. demonstrated median progression-free survival of 18.4 months in pancreatic NETs and 15.3 months in non-pancreatic NETs with CAPTEM (44). In advanced pulmonary NETs, temozolomide-based regimens achieve objective response rates of 10%–30% and median PFS of 5–13 months (41). The patient’s asymptomatic pulmonary lesion–despite bronchial involvement–aligned with CSCO guidelines recommending systemic therapy over local intervention for non-functional or rapidly progressive lung NETs (45). Our patient achieved disease stabilization for 3 months under this regimen, reflecting its moderate efficacy in controlling multifocal progression.

Bone metastases management focuses on symptomatic control and disease stabilization. Published data indicate that patients with synchronous bone metastases have reduced overall survival (OS) (46), with significantly worse outcomes compared to those with other metastatic sites (49.0 vs. 100.8 months, *p* = 0.01) (47). In a study of 85 neuroendocrine tumor patients with bone metastases, nearly two-thirds received bisphosphonates and 25.9% underwent radiation therapy (47). Another study of 74 patients reported over 70% received bone-targeting therapy (bisphosphonates, denosumab, radiotherapy, or radionuclides), with 52% achieving disease stability or better (46). In contrast, Radu et al. observed disease progression despite treatment with oxaliplatin and capecitabine (48). Our patient remained asymptomatic from skeletal lesions and received no bone-specific therapy. After three CAPTEM cycles, imaging revealed stable disease, suggesting systemic therapy alone may suffice for selected asymptomatic cases.

## Conclusion

This case exemplifies the diagnostic challenges inherent to NENs, marked by a strikingly abbreviated diagnostic interval of merely 3 months from gestational symptom onset to histopathologically confirmed diagnosis, yet accompanied by synchronous metastatic dissemination. This diagnostic paradox arises from the clinically silent progression of non-functional NETs, which evade early detection due to their indolent biological behavior and non-specific symptomatology. Optimal therapeutic navigation mandates a multidisciplinary tumor board approach, synthesizing insights from medical oncology, nuclear medicine, advanced diagnostic imaging, surgical oncology, and molecular pathology.

## Data Availability

The original contributions presented in this study are included in this article/Supplementary material, further inquiries can be directed to the corresponding author.

## Supplementary material

Supplementary Figure 1 Overview of the patient’s diagnosis and treatment timeline.
