# Supplementary figures and images for: Non-functional, non-mutated multifocal neuroendocrine neoplasms in a postpartum female: a Case Report of an infrequent disease
Source: Front Med (Lausanne). 2025 Nov 17;12:1619565. doi: 10.3389/fmed.2025.1619565 (PMC12665591; doi:10.3389/fmed.2025.1619565)

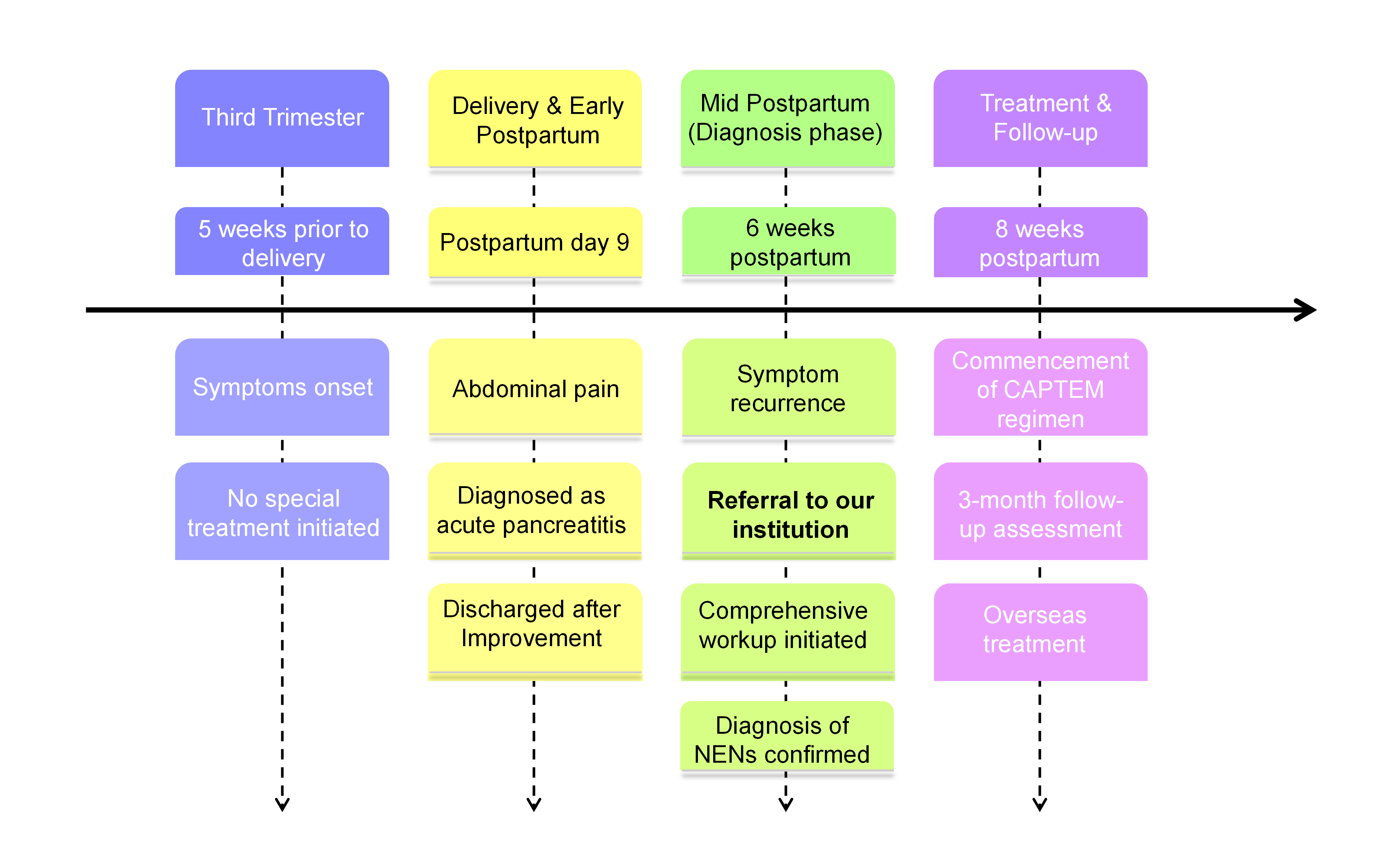

Supplement: Supplementary Figure 1 — Overview of the patient’s diagnosis and treatment timeline. [file Image_1.tif]
